# CRISPR-Cas9–Mediated TIM3 Knockout in Human Natural Killer Cells Enhances Growth Inhibitory Effects on Human Glioma Cells

**DOI:** 10.3390/ijms22073489

**Published:** 2021-03-28

**Authors:** Takayuki Morimoto, Tsutomu Nakazawa, Ryosuke Matsuda, Fumihiko Nishimura, Mitsutoshi Nakamura, Shuichi Yamada, Ichiro Nakagawa, Young-Soo Park, Takahiro Tsujimura, Hiroyuki Nakase

**Affiliations:** 1 Department of Neurosurgery, Nara Medical University, Kashihara, Nara 634-8521, Japan; nakazawa@naramed-u.ac.jp (T.N.); rmatsuda@naramed-u.ac.jp (R.M.); fnishi@naramed-u.ac.jp (F.N.); mnaka@grandsoul.co.jp (M.N.); syamada@naramed-u.ac.jp (S.Y.); nakagawa@naramed-u.ac.jp (I.N.); park-y-s@naramed-u.ac.jp (Y.-S.P.); nakasehi@naramed-u.ac.jp (H.N.); 2 Grandsoul Research Institute for Immunology, Inc., Uda, Nara 633-2221, Japan; 3 Clinic Grandsoul Nara, Uda, Nara 633-2221, Japan; takahiro@grandsoul.co.jp

**Keywords:** TIM3, CRISPR-Cas9, NK cell, glioblastoma

## Abstract

Glioblastoma (GBM) is the most common and aggressive primary malignant brain tumor in adults. Natural Killer (NK) cells are potent cytotoxic effector cells against tumor cells inducing GBM cells; therefore, NK cell based- immunotherapy might be a promising target in GBM. T cell immunoglobulin mucin family member 3 (TIM3), a receptor expressed on NK cells, has been suggested as a marker of dysfunctional NK cells. We established TIM3 knockout in NK cells, using the clustered regularly interspaced short palindromic repeats (CRISPR)-CRISPR-associated protein 9 (Cas9). Electroporating of TIM3 exon 2- or exon 5-targeting guide RNA- Cas9 protein complexes (RNPs) inhibited TIM3 expression on NK cells with varying efficacy. T7 endonuclease I mutation detection assays showed that both RNPs disrupted the intended genome sites. The expression of other checkpoint receptors, i.e., programmed cell death 1 (PD1), Lymphocyte-activation gene 3 (LAG3), T cell immunoreceptor with Ig and ITIM domains (TIGIT), and TACTILE (CD96) were unchanged on the TIM3 knockout NK cells. Real time cell growth assays revealed that TIM3 knockout enhanced NK cell–mediated growth inhibition of GBM cells. These results demonstrated that TIM3 knockout enhanced human NK cell mediated cytotoxicity on GBM cells. Future, CRISPR-Cas9 mediated TIM3 knockout in NK cells may prove to be a promising immunotherapeutic alternative in patient with GBM.

## 1. Introduction

Glioblastoma (GBM) is the most common and aggressive primary brain tumor classified as grade IV by the World Health Organization (WHO) [1]. The median overall survival (mOS) is only 15–17 months under the standard treatment consisting of surgical resection followed by radiotherapy and chemotherapy [2]. New strategies are needed for treating patients with GBM and immunotherapy can be a promising adjuvant treatment; several clinical trials have recently been reported [3,4,5].

Immunotherapy is an appealing therapy because of the potential ability of the anti-tumor effect of the immune system. Natural Killer (NK) cells were discovered more than 40 years ago and are considered to play an important role in controlling virus infections and tumor progressions to mediate cytotoxicity and produce cytokines [6,7,8]. NK cell development and maturation are dependent on the cytokine interleukin (IL)-15 [9,10,11], and are trained through the signals transduced by the activating and inhibitory receptors. While tumor-associated macrophages (TAMs) are correlated with the clinical prognosis and grade of gliomas [12], the proportion of infiltrating cytotoxic and immature NK cells is related with good prognosis [13]. However, in the brain tumor microenvironment (TME) of GBM, immunosuppression is induced to cause tumor- infiltrating lymphocyte (TIL) anergy, recruiting regulatory T cells (Treg) and activating immune checkpoints [14,15,16,17,18]. T Cell immunoglobulin mucin family member 3 (TIM3) is a member of the TIM family of receptors, of which humans have three types (TIM1, TIM3, TIM4) [19]; TIM3 is a checkpoint receptor, encoded by HAVCR2 in humans and expressed by dysfunctional CD4 and CD8 T cells [20]. TIM3 has multiple ligands (galectin 9 [21,22], phosphatidylserine (PtdSer) [20], carcinoembryonic antigen-related cell adhesion molecule 1 (CEACAM1) [23], high-mobility group protein B1 (HMGB1) [24]), which bind to different regions on the TIM3 protein. TIM3 is also expressed by NK cells, not only as a marker of effector NK cells producing Interferon (IFN) γ and undergoing degranulation [25,26], but also as a marker of dysfunction, so it is possible that TIM3 is a checkpoint receptor, when TIM3-positive NK cells encounter tumors expressing cognate ligands of TIM3 [26]. Therefore, TIM3 can be defined as a checkpoint receptor. Accordingly, TIM3 blockade reverses the dysfunction of NK cells derived from the blood of patients with melanoma [27].

We hypothesized that disrupting TIM3 in NK cells using clustered regularly interspaced short palindromic repeats (CRISPR)-CRISPR-associated protein 9 (Cas9), would improve their anti-tumor effects in GBM, similarly to the blockade or disruption of immune checkpoints such as programmed death-1 (PD-1) [28] and lymphocyte activation gene 3 (LAG3) [26,29]. Although CRISPR-Cas9 has been used with primary human T cells through retro/lentiviral delivery [30], it is difficult to infect NK cells with these viral particles. Rautela et al. reported an efficient approach to genetically editing human NK cells via electroporation and CRISPR-Cas9 ribonucleoprotein complex (RNP) [31], albeit obtaining limited amounts of genome-edited NK cells. In addition, others have reported genome-editing NK cells using induced pluripotent stem cells (iPSC), although obtaining the genome-edited NK cells is time-consuming [32]. The establishment of NK cell lines derived from patients with leukemia has also been reported [33], but the use of leukemic cell lines in the clinic is ethically problematic. We previously reported Genuine induced NK cells (GiNK), which are highly purified human NK cells derived from peripheral blood mononuclear cells (PBMCs) using a feeder-free method such as cancer cells and that exhibit high NK activity for GBM cells [34]. The expansion method can yield a large number of purified NK cells in a short time.

The specific aim of the present study was to induce TIM3 knockout in human NK cells using CRISPR-Cas9 and to evaluate the characteristics of the cells, including their growth effects on GBM cells.

## 2. Results

### 2.1. TIM3 Ligand Expression

**Figure 1:**
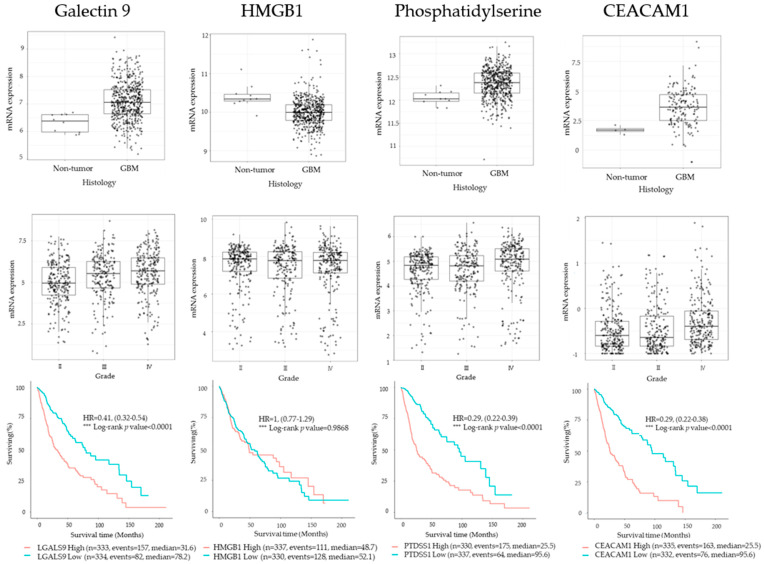
Expression of the TIM3 ligands, Galectin 9, Phosphatidylserine, HMGB1 and CEACAM1 in gliomas in TCGA data set. Top: mRNA expression of TIM3 ligands in non-tumor and GBM samples. Middle: mRNA expression of TIM3 ligands according to disease stage. Bottom: Kaplan–Meier curves based on TIM3 ligands expression.

To determine the expression pattern of the TIM3 ligands, galectin 9, PtdSer, CEACAM1, and HMGB1 in gliomas, we examined the RNA-sequencing data of gliomas from GlioVis data portal for the visualization and analysis of brain tumor expression datasets [35]. We found that all TIM3 ligands were expressed in GBM in The Cancer Genome Atlas (TCGA) database. Compared to WHO grade II and grade III glioma, GBM (grade IV) had the highest galectin 9, PtdSer and CEACAM1 expression. Further, galectin 9, PtdSer and CEACAM1 overexpression predicted significantly poorer OS in the same database (Figure 1).

**Figure 2:**
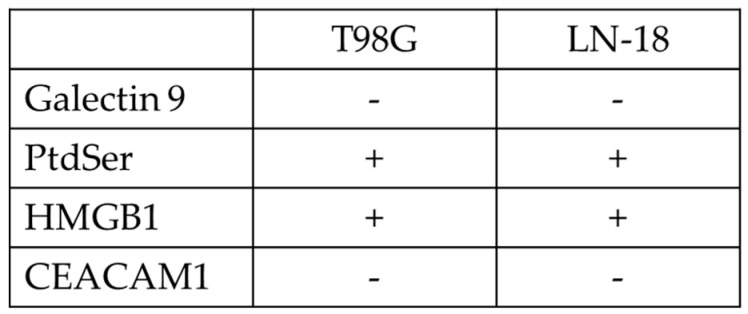
Expression of the TIM3 ligands Galectin 9, Phosphatidylserine, HMGB1 and CEACAM1 in the T98G and LN-18 glioma cell lines. Data were obtained from the Affymetrix Human Genome U133 Plus 2.0 Array (National Center for Biotechnology Information Gene Expression Omnibus database, accession no. GSE23806). Affymetrix Microarray Suite 5.0 was used to generate signal values and detection calls: absent (-) or present (+).

We also investigated TIM3 ligand expression on the GBM cell lines, T98G and LN-18 using the Affymetrix Human Genome U133 Plus 2.0 Array (National Center for Biotechnology Information Gene Expression omnibus [NCBI GEO] database [36], accession no. GSE23806). Signal values and detection calls were generated using Affymetrix Microarray Suite 5.0 (MAS5). For each probe set on each array, the MAS5 algorithm yields a detection call; Absent (A), Present (P) or Marginal (M), which indicates whether the specific mRNA is detectable. The detection call in MAS5 is based on a non-parametric statistical test (Wilcoxon signed rank test) of whether significantly more perfect matches show more hybridization signal than their corresponding mismatches [37].The results demonstrated that these two GBM cell lines expressed PtdSer and HMGB1 (Figure 2) and Detection *p* value of HMGB1 and PtdSer was 1388 and 5696 in T98G cells, was 363 and 4994 in LN18 cells.

### 2.2. Establishment of TIM3 Knockout NK Cells Using CRISPR-Cas9

**Figure 3:**
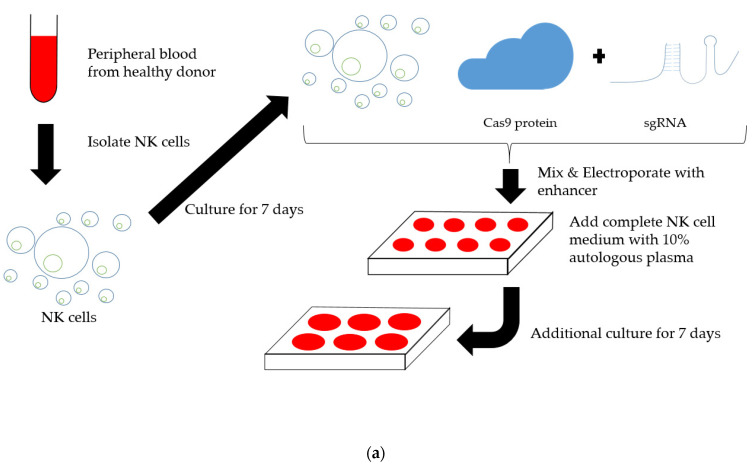

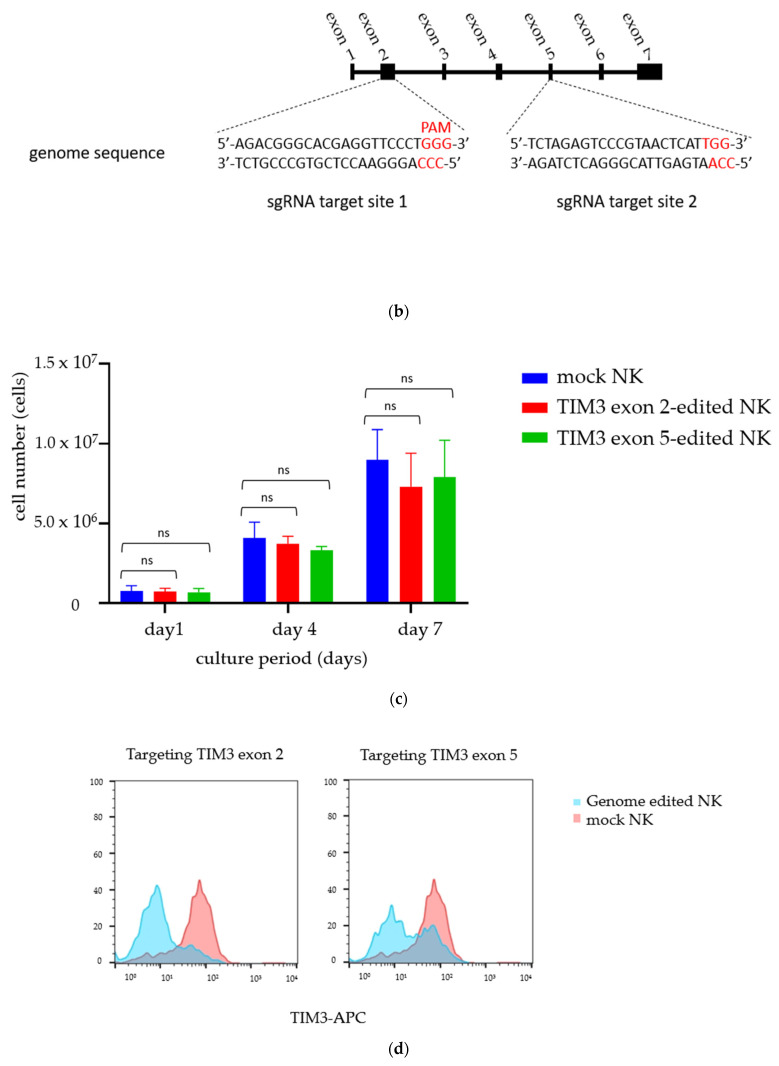

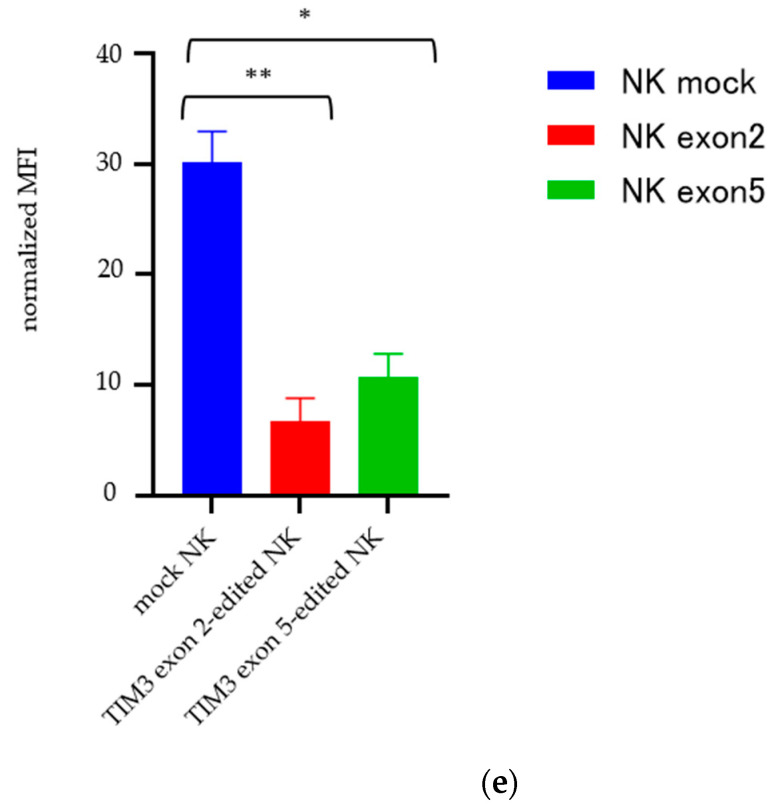
TIM3 knockout by CRISPR-Cas9 in peripheral blood mononuclear cell–derived primary human natural killer (NK) cells. (**a**) Scheme for establishing TIM3-edited human NK cells. (**b**) Schematic diagram of single guide RNAs (sgRNAs) targeting TIM3 exon 2 and exon5. Red letters indicate the proto-spacer adjacent motif (PAM) sequence. (**c**) NK cell proliferation after electroporation. Data show the mean ± standard deviation (SD). The significance of differences was determined by one-way analysis of variance (ANOVA) followed by Tukey’s test. ns: not significant. (**d**) Representative flow cytometry data of TIM3 expression on genome-edited NK cells, where TIM3 exon 2 and exon 5 were targeted. (**e**) Normalized mean fluorescence intensity (MFI) of TIM3 expression. Data show the mean ± SD of three experiments. The significance of differences was determined by one-way ANOVA followed by Tukey’s test. * *p* < 0.05, ** *p* < 0.01.

Two single-guide RNAs (sgRNAs) were designed targeting TIM3; Figure 3b show their target regions in the TIM3 gene sequence. Figure 3a shows the protocol for inducing the TIM3 knockout NK cells. We cultured primary human NK cells for 7 days after the isolating them from PBMCs obtained from a healthy volunteer. Then, RNPs created by incubating sgRNA and transactivation CRISPR RNA (tracr RNA) with recombinant Cas9 prior to electroporation, were guided into the in vitro expanded primary human NK cells. The NK cells were cultured for another 7days to allow sufficient time for protein turnover, and were stable across the duration of expansion suggesting minimal differences in the growth potential of the TIM3 knockout and mock electroporated (mock) NK cells (Figure 3c). The electroporation and transduction of CRISPR-Cas9 did not alter the NK cell growth (Figure 3c). Additionally, our NK cell expansion method induced 7.2–8.4 × 10^7^ NK cells from 16 mL human peripheral blood for 7 days’ culture. Despite the decreased viability after electroporation (25.3–27.7%), the number of cells was amplified by 15.8–27.4 times 7 days after electroporation. Ultimately, we obtained 2.9–6.4 × 10^8^ genetically modified NK cells from 16 mL blood in 2 weeks.

### 2.3. Validating TIM3 Knockout in NK Cells

**Figure 4:**
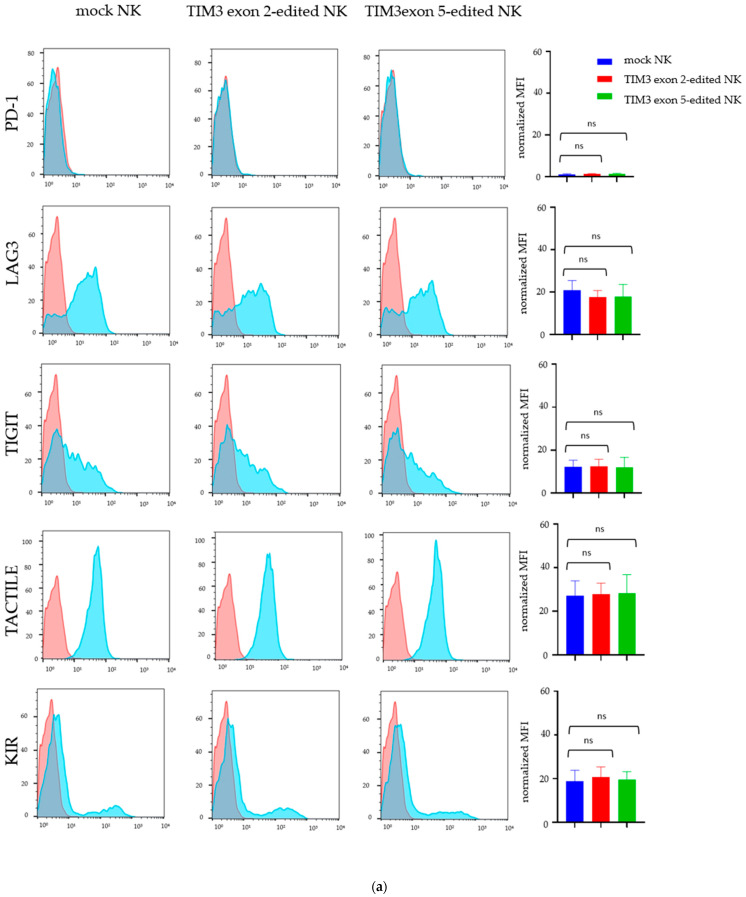

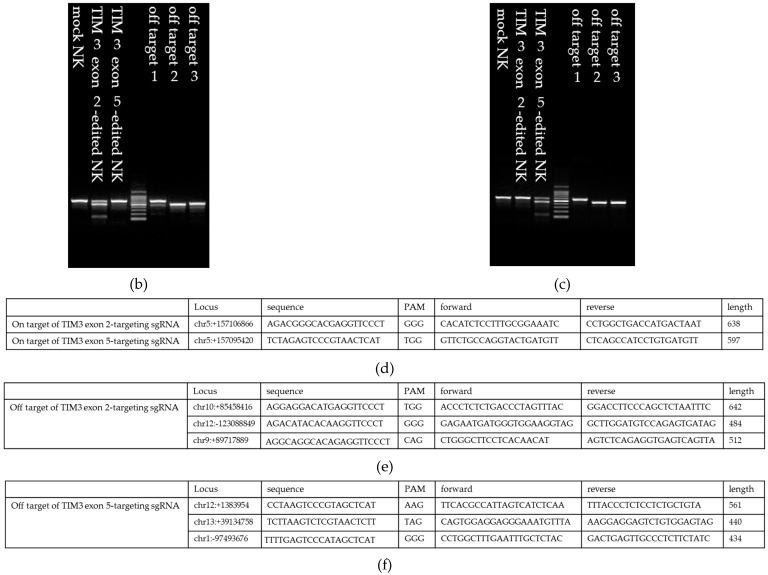
The effect of TIM3 gene knockout on the checkpoint inhibitor expression of human NK cells. The panels depict the flowcytometric data for the tested receptors on the genome-edited NK cells. (**a**) Red indicates control antibody, blue indicates genome-edited and mock NK cells. The graphs on the right show normalized MFI of the tested receptors expression. Data show the mean ± SD of four experiments. The significance of differences was determined by one-way ANOVA followed by Tukey’s test. ns: not significant. The blots show the on-target and off-target locus PCR products digested by T7E1 (T7 endonuclease I) of TIM3 knockout NK cells, targeting exon 2 (**b**) and exon 5 (**c**). The sequence shows the forward and reverse primers used in PCR for cleavage assessment by the T7E1 assays, designed for (**d**) on-target of exon 2 and exon 5, (**e**) off-target for exon 2, and (**f**) off-target for exon 5.

On day 7 after electroporation, we examined TIM3 expression in the NK cells (Figure 3d). Among the NK cell populations, the normalized mean fluorescence intensity (MFI) was 30.1 ± 1.9%, 6.7 ± 1.6%, and 10.7 ± 1.7% for the mock NK cells, TIM3 exon 2-edited NK cells, and TIM3 exon 5-edited NK cells, respectively (Figure 3e). The deletion efficacy appeared higher in the sgRNA targeting exon 2 rather than exon 5. Furthermore, the CRISPR-Cas9 system did not affect the expression of the checkpoint receptors PD1, T cell immunoreceptor with Ig and ITIM domains (TIGIT), LAG3, TACTILE (CD96), and killer inhibitory receptors (KIR); there was no significant difference between each MFI (Figure 4a).

Next, to detect on-target and off-target effects, we performed a mutation detection assay on day 7 after electroporation. The assay demonstrated clearly that both sgRNAs cleaved the TIM3 gene. Furthermore, CRISPR-Cas9 genome editing was an effective method for disrupting TIM3 on the primary human NK cells. Similarly, the T7 endonuclease I (T7E1)assays showed that the off-target mutations, predicted by the off-targeting potential detection system (Integrated DNA Technologies Inc. [IDT], Coralville, IA, USA) in the CRISPR-Cas9 system targeting exon 5 were not detected, but an off-target cleavage site was identified in the CRISPR-Cas9 system targeting exon 2 (Figure 4b,c).

### 2.4. Growth Inhibition Assay

The growth inhibitory effects of the genome-edited NK cells on the two GBM cell lines were investigated using a real-time cell analysis (RTCA system).

**Figure 5:**
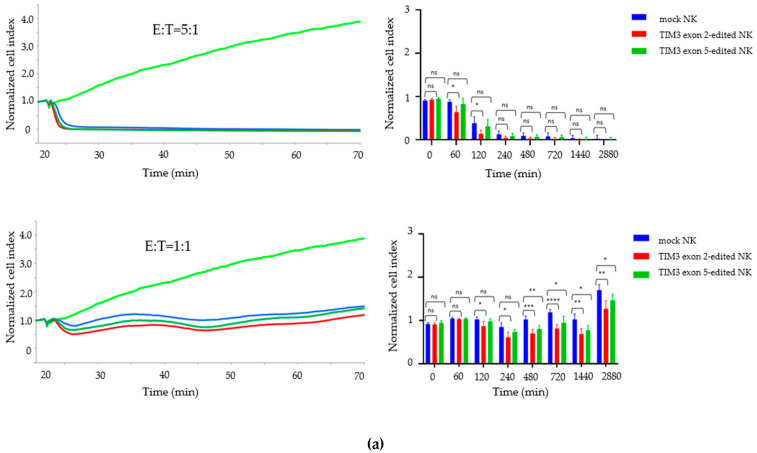

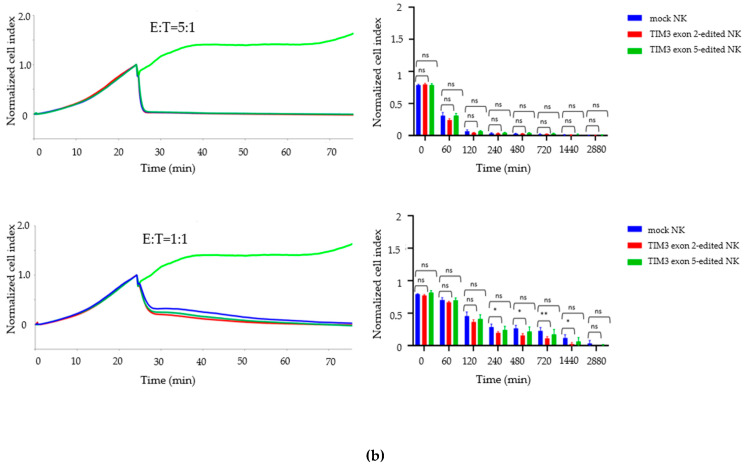
Enhanced growth inhibition of GBM cells by TIM3 knockout in NK cells. The graphs on the left show the representative growth curve of T98G (**a**) and LN-18 cells (**b**) co-cultured with mock NK cells (blue), TIM3 exon 2-edited NK cells (red), and TIM3 exon 5-edited NK cells (green) at effector-to target (E:T) cell ratios of 1:1 and 5:1. The growth curve (light green) indicates cell lines only. The graphs on the right show the real-time cell analysis based growth inhibition assays. The X- and Y-axes show the co-culture time and relative normalized cell index, respectively. Data shown are the mean ± SD of 5–6 experiments. Statistical differences were determined by two-way ANOVA followed by Tukey’s test. **** *p* < 0.0001, *** *p* < 0.001, ** *p* < 0.01, * *p* < 0.05, ns: not significant.

T98G and LN-18 cells were seeded and cultured for 1 day, then the TIM3 knockout NK cells and mock NK cells cultured for 7 days after electroporation were added to each well at an effector-to-target (E:T) cell ratio of 1:1 and 5:1. The growth inhibitory effect of TIM3 knockout NK cells on the T98G cells was clearly detected, but LN-18 cells growth was only slightly inhibited (Figure 5a,b). Compared to the mock NK cells, the TIM3 exon 2-edited NK cells significantly inhibited T98G cell growth at 2, 4, 8, 12, 24, and 48 h (E:T = 1:1) and at 2 h (E:T = 5:1), and TIM3 exon 5-edited NK cells inhibited T98G cell growth at 8, 12, 24, and 48 h (E:T = 1:1). TIM3 exon 2 edited NK cells inhibited LN-18 cell growth significantly at 4, 8, 12 and 24 h (E:T = 1:1).

## 3. Discussion

To the best of our knowledge, this is the first report to demonstrate the induction of TIM3 knockout in healthy human peripheral blood-derived NK cells by CRISPR-Cas9 electroporation. Although Rautela et al. first reported on genome-editing primary human NK cells using CRISPR-Cas9 targeting cytokine inducible SH2-containing protein (CISH) and a representative NK receptor NKp46 [31], there have been no published studies targeting TIM3. The poor infection rates of primary human NK cells with viral gene-transfer systems is a concern [31]. However, while viral pseudotyping and antagonizing the intracellular antiviral pathways have improved infection rates [38], the high risk of insertion mutations and the high cost of viral vectors for clinical therapy is a problem in lentiviral modification of human NK cells [39]. The direct delivery of the RNPs complex into human NK cells is simple and efficient means of editing the NK cell genome. In addition, the NK cell culture method we have established is suitable for gene transfer experiments because it easily yields large amount of human NK cells. Rautela et al. reported an electroporation setting of 10^5^ order of NK cells per shot: that is a small-scale validation of NK cell gene editing [31]. Our setting is 10^6^ order of NK cells per shot for electroporation. Ultimately, we were able to obtain 4.7 × 10^8^ genetically modified NK cells from 16 mL blood in 2 weeks. A clinical trial reported that a 10^8^ order infusion of cytomegalovirus-specific T cells improves the prognosis of GBM [40]. Therefore, we efficiently induced TIM3 knockout in human NK cells, and it might be possible to use it in a clinical trial.

TIM3 is a checkpoint receptor expressed by CD4 and CD8 T cells [20]. In NK cells, TIM3 is expressed by mature resting CD56^dim^ NK cells and is upregulated on activation in response to cytokine stimulation [25]. However, TIM3 blockade reverses NK cell dysfunction in NK cells derived from the PBMCs of patients with melanoma [27]. In other words, TIM3 is a marker of effector and dysfunctional NK cells. Furthermore, the function of TIM3 is dependent on the nature of the TIM3 ligand presented by the potential target cell [26]. In fact, we demonstrate that the TIM3 ligands galectin 9, PtdSer, and CEACAM1 tend to be linked to tumor grade; analysis of GlioVis data showed that their expression was highest in GBM. Patients with glioma with high galectin 9, PtdSer, and CEACAM1 expression have significantly lower survival rates than patients with low expression of these ligands.

In the present study, we designed sgRNAs against exon 2 (5′-AGACGGGCACGAGGTTCCCT-3′) and exon 5 (5′-TCTAGAGTCCCGTAACTCAT-3′) of the TIM3 gene. Flow cytometric analysis showed that TIM3 expression was clearly decreased, and therefore direct transfer of RNPs into the NK cells was feasible. We investigated other immune checkpoint receptors, i.e., PD1 [41,42], TIGIT [43,44,45], LAG3 [43], TACTILE [46,47] and KIR [48] and found no change expression between the mock NK cells and the TIM3 knockout NK cells. T7E1 mismatch detection assays confirmed that the TIM3 exon 2- and exon 5-targeting sgRNAs disrupted the intended genome sites in the NK cells. Off-target effects could not be clearly identified. The exon 2-targeting sgRNA showed an off-target effect, but the disrupted site was the intron region.

Moreover, RTCA assays revealed that CRISPR-Cas9–mediated TIM3 knockout enhanced the growth inhibitory effects of NK cells on T98G cells, which express the PtdSer and HMGB1 investigated in the NCBI GEO database. TIM3 knockout NK cells had a more significant inhibitory effect on T98G cell growth than mock NK cells. The NK cells also had enhanced growth inhibitory effects on the LN-18 cells, but the effect was less pronounced compared to that in the T98G cells. We hypothesized that it is due to the higher expression of HMGB1 and PtdSer on T98G cells than on LN-18 cells, which is demonstrated by the detection p-value calculated in the detection call in MAS5.

In summary, TIM3 knockout using the sgRNAs designed did not affect other checkpoint inhibitors, and exhibited slight off-target effects. Additionally, the growth inhibitory effects of the TIM3 exon 2 disrupted NK cells were stronger than that of the TIM3 exon 5 disruption NK cells, and flow cytometric analysis showed lower TIM3 expression in the exon 2-targeted NK cells than in the exon 5-targeted NK cells. These results demonstrate that the growth inhibitory effects of TIM3 knockout NK cells are strongly dependent on TIM3 deletion. Some reports have suggested that TIM3 is an immune checkpoint receptor depending on the context of the TIM3 ligand [25,26,49]. Our data are consistent with these reports.

Several clinical studies on GBM immunotherapy have focused on PD1 blockade, as it is a checkpoint receptor [3,4]. TIM3 also can be the novel target and blockade of TIM3 is currently being investigated in clinical trials for cancer treatment [43]. Da Silva et al. reported that TIM3 blockade improved the exhaustion of NK cells derived from patients with advanced melanoma [27]. In addition, given the expression of TIM3 ligands in GBM and NK cell infiltration [13,50], it is easy to think that the TIM3 knockout NK cells in the present study should exhibit anti-tumor effects on GBM in the clinical setting. Further, compared to antibody therapy, gene-editing NK cells has a great advantage, given the TME in GBM, where the proportion of NK cells is low and immunosuppression is high [13,50]. NK cells recruit conventional type 1 dendritic cells (cDC1) into the TME [51]. Tumor-invading TIM3 knockout NK cells might survive for a long time in the TME of GBM and elicit a systemic immune response.

The present study has some limitations. First, we evaluated the anti-tumor effect of TIM3 knockout NK cells against GBM cells in only in vitro conditions. It is necessary to investigate the anti-tumor effect of TIM3 knockout NK cells in vivo on xenografted GBM in highly immune-incompetent mice. Second, the location of the xenografted GBM differs from that of human GBM in the clinical setting; the former is an extra-axial tumor and the latter an intra-axial tumor, so the mechanism of the infiltrating immune cells may differ. On the other hand, 3D glioma cell culture [52], if possible with endothelial cells [53], could overcome the limitation of xenograft experiments. However, both xenograft model and 3D glioma model are mere attempts at reproducing human GBM microenvironment in vivo at any level, and they do not precisely reflect the human GBM microenvironment. Moreover, we strongly believe that human clinical trials should be performed first, and are preparing to do so. Finally, we used peripheral blood derived from a healthy volunteer in this study. Typically, inducing TIM3 knockout NK cells from the blood of patients with GBM is challenging because of the possibility of the patients having an immune function disorder [54]. Moreover, alkylating agents of cancer treatment such as temozolomide generally inhibit hematopoietic stem cell proliferation and limit lymphocyte numbers in the periphery [2]. It is necessary to investigate whether it is possible to induce TIM3 knockout NK cells using blood from patients with GBM.

## 4. Materials and Methods

### 4.1. Cell Lines

We obtained two standard human GBM cell lines, T98G and LN-18, from American Type Culture Collection (Manassas, VA, USA). We maintained the cells in Dulbecco’s modified Eagle’s medium (DMEM; Life Technologies, Carlsbad, CA, USA) supplemented with 10% heat-inactivated fetal bovine serum (FBS; MP Biomedicals, Tokyo, Japan), 100 U/mL penicillin, and 100 μg/mL of streptomycin (Thermo Fisher Scientific Inc., Waltham, MA, USA) at 37 °C in a humidified atmosphere containing 5% CO_2_.

### 4.2. Induction of TIM3 Knockout NK Cells

Highly purified human NK cell expansion method was performed as previously described [34]. Briefly, PBMCs were obtained from 16 mL heparinized peripheral blood obtained from a healthy volunteer (a 41-year-old man). The CD3 fraction of the PBMCs was depleted by the RosetteSep^TM^ Human CD3 Depletion Cocktail (STEMCELL Technologies, Vancouver, Canada). The CD3-depleted PBMCs were placed in a T25 culture flask (Corning, Steuben, NY, USA) containing AIM V medium (Life Technologies) supplemented with 10% autologous plasma, 50 ng/mL recombinant human IL-18 (rhIL-18, Medical & Biological Laboratories Co., Ltd., Nagoya, Japan), and 3000 IU/mL rhIL-2 (Novartis, Basel, Switzerland) at 37 °C in a humidified atmosphere containing 5% CO_2_ for 7 days. The AIM V medium containing 3000 IU/mL rhIL-2 was replenished as necessary. Then, 3 × 10^6^ of the expanded NK cells were electroporated to RNPs complexes targeting TIM3 using an Amaxa Human NK cell Nucleofector Kit (VPA-1005; Lonza, Basel, Switzerland) and electroporation program X-001. Subsequently, the cells were resuspended in AIM V medium containing 10% autologous plasma and 3000 IU/mL rhIL-2 and placed in a 12-well plate (Corning) at 37 °C in a humidified atmosphere containing 5% CO_2_ for 7 days.

### 4.3. Antibody Staining and Flow Cytometry

The cells were stained with the appropriate antibodies, and fixed with 1% paraformaldehyde-containing phosphate-buffered saline (PBS) at 4 °C for 1 h. Data were obtained using a BD FACSCalibur flow cytometer (BD Biosciences, San Jose, CA, USA) and analyzed using FlowJo, version 10 (BD Biosciences). The following antibodies were used: Alexa 488-labeled anti-CD56 (clone B159, BD Pharmingen, New Jersey, USA), Allophycocyanin (APC)-labeled anti-PD-1 (EH12.2H7, Bio Legend, San Diego, CA, USA), APC-labeled anti-TIM-3 (F38-2E2, Miltenyi Biotech, Tokyo, Japan), APC-labeled anti-LAG-3 (CD223) (REA351, Miltenyi Biotech), APC-labeled anti-TIGIT (REA1004, Miltenyi Biotech), APC-labeled anti-TACTILE (REA195, Miltenyi Biotech) and APC-labeled anti-KIR2DL1 (CD158a) (REA284, Miltenyi Biotech).

### 4.4. Designing the sgRNAs

We prepared two sgRNAs targeting the exon 2 (5′-AGACGGGCACGAGGTTCCCT-3′) and exon 5 regions (5′-TCTAGAGTCCCGTAACTCAT-3′) of the human TIM3 gene according to the manufacturer’s instructions (IDT, https://sg.idtdna.com/site/order/designtool/index/CRISPR_PREDESIGN).

### 4.5. Gene Deletion Efficacy of CRISPR-Cas9

The genome-edited cells were harvested 7 days after the electroporation, and their DNA was extracted with a QIA amp DNA mini kit (Qiagen, Hilden, Germany) and subjected to T7E1 mismatch detection assays using an Alt-R Genome Editing Detection Kit (IDT). The on-target and off-target sites and adjacent sequences were amplified from the genomic DNA using KOD FX enzyme solution (TOYOBO, Osaka, Japan). The PCR primers are listed in Figure 4d–f. The PCR conditions were as follows: one cycle at 94 °C for 2 min, followed by 40 cycles at 98 °C for 10 s, 63 °C for 30 s, and 68 °C for 30 s, and finally one cycle at 68 °C for 7 min. PCR was performed using a Life ECO thermal cycler (Bioer Technologies Co. Ltd., Hangzhou, China). The PCR primer sequences were obtained from Thermo Fischer Scientific. The amplicons from the TIM3 exon 2 and exon 5 primers were 638-bp and 597-bp long, respectively. Then, the PCR was run using the Life ECO thermal cycler with the following cycling conditions: 95 °C for 5min, decrease from 95 °C to 85 °C, at a rate of 2 °C per second, decrease from 85 °C to 25 °C at a rate of 0.1 °C per second, and decrease to 4 °C. The rehybridized PCR products were digested with T7E1 for 30 min and separated on 2% agarose gel for 20 min. The DNA was visualized under a UV transilluminator (FAS-IV; NIPPON Genetics, Kyoto, Japan). In the same way, off-target mutagenesis, predicted with an off-targeting potential checking system (IDT, https://sg.idtdna.com/site/order/designtool/index/CRISPR_PREDESIGN), was detected; the PCR primers used for amplifying the target locus are listed in Figure 4d–f.

### 4.6. Growth Inhibition Assays

The inhibitory effects of the genome-edited NK cells on GBM cells were investigated using the xCELLigence RTCA S16 and DP instruments (ACEA Biosciences, San Diego, CA, USA). The procedure has been described previously [55]. Briefly, complete medium (100 μL) was added to each well on E-plate 16 (ACEA Biosciences), and background impedance was measured at 37 °C in a humidified atmosphere containing 5% CO_2_. The GBM cells, i.e., 2 × 10^4^/well T98G or LN-18cells, were seeded in each well as the target cells, and impedance measurement was recorded every 5 min for 72 h. After 24 h, the genome-edited NK cells were added to each well as the effector cells in the defined E:T cell ratios. The data were analyzed using RTCA, version1.2 (ACEA Biosciences).

### 4.7. Statistical Analysis

Statistical analyses were performed using Prism 8 (GraphPad Software Inc., San Diego, CA, USA). The data are shown as the mean ± standard deviation (SD). The statistical significance of differences was determined using the one-way or two-way analysis of variance (ANOVA) followed by Turkey’s test. We considered *p*-values < 0.05 as statistically significant.

## 5. Conclusions

We successfully performed CRISPR-Cas9–mediated TIM3 knockout in human primary NK cells and demonstrated their growth inhibitory effect on GBM cell lines without altering the expression of other checkpoint receptors. The data clearly reveal that TIM3 plays an important role as an immune checkpoint receptor that inhibits human NK cell function. In the future, TIM3 knockout NK cells obtained via direct transfer of the CRISPR-Cas9 protein complex might be promising therapeutic designer NK cells for treating patients with GBM.

## Data Availability

The data that support the findings of this study are available from the corresponding author upon reasonable request.
